# Supplementary material for: The Effects of Outdoor versus Indoor Exercise on Psychological Health, Physical Health, and Physical Activity Behaviour: A Systematic Review of Longitudinal Trials
Source: Int J Environ Res Public Health. 2023 Jan 17;20(3):1669. doi: 10.3390/ijerph20031669 (PMC9914639; doi:10.3390/ijerph20031669)
Supplement: Supplementary file 1 [file ijerph-20-01669-s001.zip › ijerph-2084503-supplementary.pdf]

Table S1. PRISMA 2020 Checklist.

Section and Topic	Item #	Checklist item	Location where item is reported
**TITLE**			
Title	1	Identify the report as a systematic review.	1
**ABSTRACT**			
Abstract	2	See the PRISMA 2020 for Abstracts checklist.	1
**INTRODUCTION**			
Rationale	3	Describe the rationale for the review in the context of existing knowledge.	1–2
Objectives	4	Provide an explicit statement of the objective(s) or question(s) the review addresses.	2
**METHODS**			
Eligibility criteria	5	Specify the inclusion and exclusion criteria for the review and how studies were grouped for the syntheses.	3–4
Information sources	6	Specify all databases, registers, websites, organisations, reference lists and other sources searched or consulted to identify studies. Specify the date when each source was last searched or consulted.	3
Search strategy	7	Present the full search strategies for all databases, registers and websites, including any filters and limits used.	3 & Table S2
Selection process	8	Specify the methods used to decide whether a study met the inclusion criteria of the review, including how many reviewers screened each record and each report retrieved, whether they worked independently, and if applicable, details of automation tools used in the process.	3
Data collection process	9	Specify the methods used to collect data from reports, including how many reviewers collected data from each report, whether they worked independently, any processes for obtaining or confirming data from study investigators, and if applicable, details of automation tools used in the process.	4
Data items	10a	List and define all outcomes for which data were sought. Specify whether all results that were compatible with each outcome domain in each study were sought (e.g. for all measures, time points, analyses), and if not, the methods used to decide which results to collect.	4
	10b	List and define all other variables for which data were sought (e.g. participant and intervention characteristics, funding sources). Describe any assumptions made about any missing or unclear information.	4
Study risk of bias assessment	11	Specify the methods used to assess risk of bias in the included studies, including details of the tool(s) used, how many reviewers assessed each study and whether they worked independently, and if applicable, details of automation tools used in the process.	4–5
Effect measures	12	Specify for each outcome the effect measure(s) (e.g. risk ratio, mean difference) used in the synthesis or presentation of results.	4
Synthesis methods*	13a	Describe the processes used to decide which studies were eligible for each synthesis (e.g. tabulating the study intervention characteristics and comparing against the planned groups for each synthesis (item #5)).	5
	13b	Describe any methods required to prepare the data for presentation or synthesis, such as handling of missing summary statistics, or data conversions.	NA
	13c	Describe any methods used to tabulate or visually display results of individual studies and syntheses.	5
	13d	Describe any methods used to synthesize results and provide a rationale for the choice(s). If meta-analysis was performed, describe the model(s), method(s) to identify the presence and extent of statistical heterogeneity, and software package(s) used.	5

Table S1. *Cont.*

Section and Topic	Item #	Checklist item	Location where item is reported
	13e	Describe any methods used to explore possible causes of heterogeneity among study results (e.g. subgroup analysis, meta-regression).	NA
	13f	Describe any sensitivity analyses conducted to assess robustness of the synthesized results.	NA
Reporting bias assessment	14	Describe any methods used to assess risk of bias due to missing results in a synthesis (arising from reporting biases).	4
Certainty assessment	15	Describe any methods used to assess certainty (or confidence) in the body of evidence for an outcome.	NA
**RESULTS**			
Study selection	16a	Describe the results of the search and selection process, from the number of records identified in the search to the number of studies included in the review, ideally using a flow diagram.	5–6
	16b	Cite studies that might appear to meet the inclusion criteria, but which were excluded, and explain why they were excluded.	6
Study characteristics	17	Cite each included study and present its characteristics.	6–9 & Table S3
Risk of bias in studies	18	Present assessments of risk of bias for each included study.	9–11
Results of individual studies	19	For all outcomes, present, for each study: (a) summary statistics for each group (where appropriate) and (b) an effect estimate and its precision (e.g. confidence/credible interval), ideally using structured tables or plots.	Table S4
Results of syntheses*	20a	For each synthesis, briefly summarise the characteristics and risk of bias among contributing studies.	NA
	20b	Present results of all statistical syntheses conducted. If meta-analysis was done, present for each the summary estimate and its precision (e.g. confidence/credible interval) and measures of statistical heterogeneity. If comparing groups, describe the direction of the effect.	11–14
	20c	Present results of all investigations of possible causes of heterogeneity among study results.	NA
	20d	Present results of all sensitivity analyses conducted to assess the robustness of the synthesized results.	NA
Reporting biases	21	Present assessments of risk of bias due to missing results (arising from reporting biases) for each synthesis assessed.	10
Certainty of evidence	22	Present assessments of certainty (or confidence) in the body of evidence for each outcome assessed.	NA
**DISCUSSION**			
Discussion	23a	Provide a general interpretation of the results in the context of other evidence.	17 (4.4)
	23b	Discuss any limitations of the evidence included in the review.	16 (4.2)
	23c	Discuss any limitations of the review processes used.	15–16 (4.3)
	23d	Discuss implications of the results for practice, policy, and future research.	16–17 (4.5)
**OTHER INFORMATION**			
Registration and protocol	24a	Provide registration information for the review, including register name and registration number, or state that the review was not registered.	2
	24b	Indicate where the review protocol can be accessed, or state that a protocol was not prepared.	18

Table S1. *Cont.*

Section and Topic	Item #	Checklist item	Location where item is reported
	24c	Describe and explain any amendments to information provided at registration or in the protocol.	2
Support	25	Describe sources of financial or non-financial support for the review, and the role of the funders or sponsors in the review.	17
Competing interests	26	Declare any competing interests of review authors.	18
Availability of data, code and other materials	27	Report which of the following are publicly available and where they can be found: template data collection forms; data extracted from included studies; data used for all analyses; analytic code; any other materials used in the review.	18

*For items 13, 15, 20, and 22, many elements are not applicable to the present systematic review because meta-analyses were not possible.

Table from: Page, M.J.; McKenzie, J.E.; Bossuyt, P.M.; Boutron, I.; Hoffmann, T.C.; Mulrow, C.D.; Shamseer, L.; Tetzlaff, J.M.; Akl, E.A.; Brennan, S.E.; et al. The PRISMA 2020 Statement: An Updated Guideline for Reporting Systematic Reviews. *BMJ* **2021**, *372*, doi:10.1136/BMJ.N71.

Table S2. Search strategies for all databases, searched on March 23, 2021, with no time restrictions.

Databases: MEDLINE and Epub Ahead of Print, In-Process, In-Data-Review & Other Non-Indexed Citations, Daily and Versions; Cochrane Central Register of Controlled Trials (CENTRAL); and Embase, all via Ovid.

Search Terms:

1. (green exercis* or green gym* or blue exercis* or blue gym* or ecotherap*).ti,ab.
2. (exercis* or physical activit* or walk* or physical fit* or run* or athlet*).ti,ab.
3. (outdoor* or outside* or park* or greenspace* or green space* or bluespace* or blue space* or natural environment* or nature or forest* or biodivers* or horticultur*).ti,ab
4. (indoor* or inside* or laboratory or gym* or home* or buil*).ti,ab.
5. 2 and 3
6. 1 or 5
7. 4 and 6
8. limit 7 to english language

Databases: APA PsycINFO; CINAHL Complete; GreenFile; and SPORTDiscus, all via EBSCOhost

Search Terms:

1. green exercis* OR green gym* OR blue exercis* OR blue gym* OR ecotherap*
2. (exercis* OR physical activit* OR walk* OR physical fit* OR run* OR athlet*) AND (outdoor* OR outside* OR park* OR greenspace* OR green space* OR bluespace* OR blue space* OR natural environment* OR nature OR forest* OR biodivers* OR horticultur*)
3. 1 or 2
4. indoor* OR inside* OR laboratory OR gym* OR home* OR buil*
5. 3 and 4

Language Limiter: English

Database: PubMed

Search Terms:

1. ((exercis*[Title/Abstract] OR physical activit*[Title/Abstract] OR walk*[Title/Abstract] OR physical fit*[Title/Abstract] OR run*[Title/Abstract] OR athlet*[Title/Abstract]) AND (outdoor*[Title/Abstract] OR outside*[Title/Abstract] OR park*[Title/Abstract] OR greenspace*[Title/Abstract] OR green space*[Title/Abstract] OR bluespace*[Title/Abstract] OR blue space*[Title/Abstract] OR natural environment*[Title/Abstract] OR nature[Title/Abstract] OR forest*[Title/Abstract] OR biodivers*[Title/Abstract] OR horticultur*[Title/Abstract])) OR (green exercis*[Title/Abstract] OR green gym*[Title/Abstract] OR blue exercis*[Title/Abstract] OR blue gym*[Title/Abstract] OR ecotherap*[Title/Abstract])) AND (indoor*[Title/Abstract] OR inside*[Title/Abstract] OR laboratory[Title/Abstract] OR gym*[Title/Abstract] OR home*[Title/Abstract] OR buil*[Title/Abstract]) AND English[Language]

Database: Web of Science

Search Terms:

1. TS=(green exercis* OR green gym* OR blue exercis* OR blue gym* OR ecotherap*)
2. TS=(exercis* OR physical activit* OR walk* OR physical fit* OR run* OR athlet*)
3. TS=(outdoor* OR outside* OR park* OR greenspace* OR green space* OR bluespace* OR blue space* OR natural environment* OR nature OR forest* OR biodivers* OR horticultur*)
4. 2 and 3
5. 1 or 4
6. TS=(indoor* OR inside* OR laboratory OR gym* OR home* OR buil*)
7. 5 and 6
8. Language: (English)

Table S3. Characteristics of included studies. Acronyms: NR: not reported. OE: Outdoor Exercise group. IE; Indoor Exercise group. Sample size (N) refers to total number of participants allocated to OE and IE groups at baseline, unless otherwise noted. Age shown in years (y) as mean ± standard deviation.

Author, Date Location, Setting	Study Design	Participants	Exercise Environment		Exercise Protocol		Duration	Outcomes (measure)
			Outdoors	Indoors	Outdoors	Indoors		
Abdel-Rahman and Magdy, 2014<br><br>Egypt, setting NR	Between subjects, controlled trial, randomization NR, parallel group	Professional Epee fencers from Egyptian fencing clubs<br><br>N=20 (10 per group)<br><br>Age (y): OE: 21.20 ± 1.6; IE: 20.11 ± 1.9<br><br>Sex NR	Outdoors	"Into the hall"	"Warm-up, stretch, and body weight exercises."	"Traditional training"<br>Note: "Only the part of warm-up [was] different between the two groups."	8 weeks, frequency NR	(Test of Performance Strategies)<br>• Goal Setting<br>• Self Talk<br>• Relaxation<br>• Automacity<br>• Activation<br>• Emotional Control<br>• Imagery<br>• Attentional Control<br>• Negative Thinking<br>Flexibility (Sit and reach test)<br>Agility (Shuttle run)<br>Counter time (during fencing)
Anandh, Varadharajulu, & Alate, 2020<br><br>India, University	Between subjects, controlled trial, randomization NR, parallel group	Asymptomatic, retired seniors, restricted to their residence most of the time<br><br>N=76<br><br>Age (y): >65<br><br>Sex NR; "Both genders"	"Outdoor (open) natural environment without risky additional barriers like sloppy surface, uneven terrain, traffic areas, noisy environment, etc.,"	"Indoor (closed) environment"	60 min exercise program including warm-up & cool-down: 1. Jacobson's relaxation: 5 min. 2. Free exercises in extremities and spine: 5 min. 3. Personal activities (Folding Bed sheet/counting currency & Dressing self): 10 min. 4. Cognitive exercises (Loud reading, Writing/Drawing while listening to music & Brushing teeth with non-dominant hand): 10 min. 5. Dual task activity: 20 min (including reading/talking while walking: 5	60 min exercise program including warm-up & cool-down: 1. Jacobson's relaxation: 5 min. 2. Free exercises in extremities and spine: 5 min. 3. Personal activities (Folding Bed sheet/counting currency & Dressing self): 10 min. 4. Cognitive exercises (Loud reading, Writing/Drawing while listening to music & Brushing teeth with non-dominant hand): 10 min. 5. Dual task activity: 20 min (including reading/talking while walking: 5	2 x about 60 min sessions per week for 12 weeks	Social Activity Self-Efficacy<br>Timed 10-Meter Walk Test<br>Timed Up and Go Test

					min; follow the light/cues while walking: 5 min; obstacle walking: 10 min). 6. Recreational activity (catching & throwing ball): 5 min. 7. Relax breathing exercises: 5 min.	min; follow the light/cues while walking: 5 min; obstacle walking: 10 min). 6. Recreational activity (catching & throwing ball): 5 min. 7. Relax breathing exercises: 5 min.		
Calogiuri, Nordtug, & Weydahl, 2015 [Intervention study]<br><br>Norway, Workplace	Between subjects, randomized controlled trial, parallel group	Healthy employees from 2 workplaces<br><br>N=14<br><br>Age (y): 48.5 ± 7.3<br><br>Sex: 50% male, 50% female<br><br>BMI (kg/m^2): 25.20 ± 2.47	Nature area near workplace with trees and grass and few built elements.<br><br>Biking along a 6082 m track on participants' own bikes in a forest area nearby both workplaces, with a fairly even terrain and one steep uphill section (200-m long, 25-m height difference).<br><br>Temperature: 8–10°C<br>Month: September<br>Weather: excellent or overcast	Gym hall, ventilated, well illuminated by artificial light and white curtain-covered windows, and a large mirror<br><br>Temperature: 20°C<br><br>Humidity: 60%	Two sessions of 25 min outdoor biking (5 min warm-up, 15 min workout, 5 min cool-down), and 20 min strength circuit (8 exercises covering all major muscle groups) using elastic rubber bands with handles.<br><br>Moderate intensity (Borg RPE 12–13) in warm-up and cool-down and moderately high intensity (Borg RPE 15–16) during workout.	Two sessions of 25 min biking (5 min warm-up, 15 min workout, 5 min cool-down) on stationary bikes with 1.5 min bench step-ups to mimic outdoor uphill section, and 20 min strength circuit (8 exercises covering all major muscle groups) using elastic rubber bands with handles.<br><br>Moderate intensity (Borg RPE 12–13) in WU and CD and moderately high intensity (Borg RPE 15–16) during WO.	2 x about 50-min sessions, 2 days apart; 2- and 10-week follow-up	(Perceived Restorativeness Scale)<br>• Fascination<br>• Being Away<br>Enjoyment (0–10 scale)<br>• Biking<br>• Strength<br>Future exercise intention (3-item scale)<br>Perceived exertion (Borg 6–20 RPE)<br>Exercise intensity (%HRR)<br>Affect (Physical Activity Affective Scale):<br>• Positive Affect<br>• Negative Affect<br>• Tranquility<br>• Fatigue<br>Follow-up exercise behavior (Leisure Time Exercise Questionnaire)<br>Follow-up exercise behavior (1-3 scale):<br>• Biking indoors<br>• Biking in nature<br>• Exercising with rubber bands<br>Qualitative responses to exercise environment
Calogiuri *et al.*, 2016<br><br>Norway, University	Between subjects, randomized controlled	Healthy employees from 2 workplaces, sedentary or	Biking along a 6082 m track on participants' own bikes	"Typical exercise setting (gym hall)", no visual contact with nature,	Two sessions of 25-min biking and 20-min strength circuit (8 exercises covering all major	Two sessions of 25-min stationary cycling (including bench step-ups to reproduce	2 x 45-min sessions on different days over 2-week span; 2- and	(Perceived Restorativeness Scale)<br>• Fascination<br>• Being Away

	trial, parallel group	moderately active; office-based occupations<br><br>N=14 (7 per group)<br><br>Age (y): 49 ± 8<br><br>Sex: 50% male, 50% female<br><br>BMI (kg/m²): - 25.20 ± 2.47	in a forest area nearby both workplaces, with a fairly even terrain and one steep uphill section (200-m long, 25-m height difference).<br><br>Strength session in a grass yard of one of the workplaces just outside the forest area.<br><br>Temperature: 8–10°C<br>Month: September<br>Weather: sunny or overcast	well illuminated with artificial light and natural light through white curtain-covered windows, and a large mirror<br><br>Temperature: 20°C<br><br>Humidity: 60%<br><br>Month: September	muscle groups) using elastic rubber bands with handles.<br><br>Intensity: about 55% heart rate reserve, overall; instructed to maintain moderate intensity during warm-up and cool-down, and moderately high intensity during workout (Borg scale)	outdoor uphill section) and 20-min strength circuit (8 exercises covering all major muscle groups) using elastic rubber bands with handles.<br><br>Intensity: about 55% heart rate reserve, overall; instructed to maintain moderate intensity during warm-up and cool-down, and moderately high intensity during workout (Borg scale)	10-week follow up measurements on same days in both groups	Affect (Physical Activity Affective Scale):<br>• Positive Affect<br>• Negative Affect<br>• Tranquility<br>• Fatigue<br>Systolic BP<br>Diastolic BP<br>Cortisol (Salivary cortisol awakening response; Serum cortisol concentration)<br>Follow-up Affect (Physical Activity Affective Scale):<br>• Positive Affect<br>• Tranquility
Duranso, 2018*<br><br>USA, University	Between subjects, randomized controlled trial, parallel group	Full-time undergraduate college students; sedentary at baseline<br><br>N=87 (OE: N=48; IE: N=39) completers<br><br>Age (y) for post-test parent sample: 19 (range: 18–22)<br><br>Sex for post-test parent sample: 20.5% male, 77.3% female, 2.2% NR	"Outdoors"	"Indoors"	Walk briskly outdoors, alone, at a walking pace that raised heart rate and breathing and could be maintained for the entire 30 min.<br>Track exercise with activity log (day, time, duration, context) and Mapmywalk smart phone application.<br>Avoid walking at night or with headphones.	Walk briskly indoors, alone, at a walking pace that raised heart rate and breathing and could be maintained for the entire 30 min.<br>Track exercise with activity log (day, time, duration, context).	≥ 30 min x 4 sessions per week for 8 weeks	Exercise-specific approach motivation (Achievement Goals Questionnaire for Sport)<br>Exercise-specific self-efficacy (Exercise Self-Efficacy Scale)<br>General self-efficacy (New Generalized Self-Efficacy Scale)<br>Global approach motivation (revised Appetitive Motivation Scale)

Irandoust & Taheri, 2017§<br><br>Iran, setting NR	Between subjects, randomized controlled trial, parallel group	Obese, severely depressed women with vitamin D deficiency<br><br>N=30 (15 per group)<br><br>Age (y) for parent sample: 43.2 ± 12.4<br><br>BMI (kg/m²): 30.0-35.0	Outdoors<br><br>Date: Fall 2015, 11:00–12:00am	On a treadmill at a health club<br><br>Date: Fall 2015, 11:00–12:00am	5 min warm-up stretching, 50 min jogging, 5 min cool-down stretching; at a target heart rate of 55%–75% (55% for weeks 1 and 2, 65% for weeks 3 and 4, and 75% for weeks 5–12) "based on the Karvonen method", and RPE 11–12	5 min warm-up stretching, 50 min jogging, 5 min cool-down stretching; at a target heart rate of 55%–75% (55% for weeks 1 and 2, 65% for weeks 3 and 4, and 75% for weeks 5–12) "based on the Karvonen method", and RPE 11–12	4 x 1-hour sessions, 4 times per week for 3 months	Depression (Beck Depression Inventory)<br>Weight<br>BMI<br>Waist to hip ratio<br>% Body fat<br>Serum 25(OH) vitamin D<br>Food behaviour
Lacharité-Lemieux, Brunelle, and Dionne, 2015<br><br>Canada, University-associated Research Centre on Aging	Between subjects, randomized controlled trial, parallel group	Sedentary, post-menopausal females, white, non-smoking, healthy<br><br>N=23<br><br>Age (y): 60.7 ± 4.8	A "mainly natural park beside body of water", with "paths lined with large trees and rich biodiversity"; static aerobic part in middle of park, in an open green area surrounded by river and old pine trees; large tent in center of park used when heavy rain<br><br>Date: April to July 2013	A large meeting room in Research Center on Aging; carpeted floor, many windows with view of parking lot, heated to same temperature as outdoor<br><br>Date: April to July 2013	1-hour sessions of mixed training: 10 min dynamic aerobic training; 20 min static aerobic circuit (elastics, dumbbells, and steps); 15 min resistance training (2-3 sets of 12-15 reps using elastics and mats); cool-down<br><br>Intensity: instructed to maintain target heart rate progressing every 3 weeks from 65-95% of maximum	1-hour sessions of mixed training: 10 min dynamic aerobic training; 20 min static aerobic circuit (elastics, dumbbells, and steps); 15 min resistance training (2-3 sets of 12-15 reps using elastics and mats); cool-down<br><br>Intensity: instructed to maintain target heart rate progressing every 3 weeks from 65-95% of maximum	3 x 1-hour sessions per week (Mon, Wed, Fri) for 12 weeks	Affective Valence (Feeling Scale)<br>Affective Activation (Felt Arousal Scale)<br>(Exercise-Induced Feeling Inventory)<br>• Positive engagement<br>• Revitalization<br>• Physical exhaustion<br>• Tranquility<br>Exercise adherence (% sessions attended)<br>Weight<br>BMI<br>Physical activity level (Physical Activity Scale for the Elderly)<br>Depression (Beck Depression Inventory)<br>Perceived exertion (Borg 6-20 RPE)
Lacharité-Lemieux & Dionne, 2016<br><br>Canada, University-associated Research	Between subjects, randomized controlled trial, parallel group	Sedentary, post-menopausal females, white, non-smoking, healthy<br><br>N=23	"Aesthetically pleasing and mainly natural park beside a body of water, where paths were lined with large trees and rich biodiversity"	Large meeting room in the Research Center on Aging; carpeted floor, many windows with a view of the parking lot on both sides	1-hour sessions of mixed training: 10 min dynamic aerobic training; 20 min static aerobic circuit (elastics, dumbbells, and steps); 15 min resistance training	1-hour sessions of mixed training: 10 min dynamic aerobic training; 20 min static aerobic circuit (elastics, dumbbells, and steps); 15 min resistance training	3 x 1-hour sessions per week (Mon, Wed, Fri) for 12 weeks	Self-chosen intensity, mean and maximal (absolute and relative HR)<br>Weight<br>Waist circumference<br>BMI<br>Fat mass<br>Lean mass<br>% Fat mass

Centre on Aging		Age (y): 60.7 ± 4.8	Date: April to July 2013	Date: April to July 2013	(2-3 sets of 12-15 reps using elastics); cool-down<br><br>Self-selected intensity: “an intensity of preference that can be sustained for [35 minutes] and that they would feel happy to do regularly” (Parfitt, Rose, & Markland, 2000).	(2-3 sets of 12-15 reps using elastics); cool-down<br><br>Self-selected intensity: “an intensity of preference that can be sustained for [35 minutes] and that they would feel happy to do regularly” (Parfitt, Rose, & Markland, 2000).		Muscle mass index (DXA)<br>VO_{2max} (modified Balke test)<br>Plasma lipids: triglycerides; total cholesterol; cholesterol–LDL; cholesterol–HDL<br>Fasting glucose<br>Fasting insulin<br>HOMA-IR<br>Diastolic BP<br>Systolic BP<br>Muscle strength (1 rep max, 1-RM)<br>• Leg press<br>• Bench press<br>• Lat pulldown<br>Muscle endurance (Max reps at 70% -RM):<br>• Leg press<br>• Bench press<br>• Lat pulldown
Miller *et al.*, 2020<br><br>USA, University	Within subjects, cluster-randomized controlled trial, crossover (2 groups)	Childhood cancer survivors from Minnesota<br><br>N=19 (14 analyzed)<br><br>Age (y): 19.7 (95% CI: 13.3–27.6)<br><br>Sex: 47.4% male, 52.6% female<br><br>Mean moderate-to-vigorous physical activity (min/week): 55.4 ± 25.7	A large park in Minneapolis<br><br>Date: June and July 2019	In the tunnels and skyways at the University of Minnesota<br><br>Date: June and July 2019	30-50 min walking sessions, paced by peer leader. Participants were allowed to bring a friend or family member with them and were encouraged to socialize.	30-50 min exercise sessions, paced by peer leader. Participants were allowed to bring a friend or family member with them and were encouraged to socialize.	1-2 x 30-50 min sessions in 1 environment during 1 week in June 2019; 1-2 x 30-50 min sessions in the other environment during 1 week in July 2019	Exercise motivation (Behavioral Regulation in Exercise Questionnaire-2):<br>• Overall Score<br>• Intrinsic<br>• Identified<br>• Introjected<br>• Extrinsic<br>• Amotivation<br>(Psychological Need Satisfaction in Exercise):<br>• Autonomy<br>• Competence<br>• Relatedness<br>Moderate to vigorous physical activity:<br>• Habitual (weekly)<br>• In-session<br>Fatigue (Fatigue scale-adolescent; FSA):<br>• General<br>• In-session

Moslehi, Moslehi, & Khalvati, 2019<br><br>Iran, setting NR	Between subjects, randomized controlled trial, parallel group	Obese adolescent schoolboys<br><br>N=20 (10 per group)<br><br>Age (y): POAE: 11.12 ± 0.51; ITAE: 10.87 ± 0.54	On a 20 × 40 m artificial turf football field; "a temperate temperature of 21–24 °C, a height of 1870 m above sea level, a moderate forest cover, gentle wind blowing and the training was done in the morning under the sun shine with 50–60% humidity."	In a closed area with 12 m in length, 8 m in width and 5 m in height dimensions, 1870 m above sea level at 8–11 a.m. in 21–24°C and 50% humidity on the treadmill device Model T9300 turbo manufactured in Taiwan.	Playing in the form of Outdoor Aerobic Exercise (POAE): warm-up at 20% reserve heart rate (HRR); aerobic exercise in the form of a football game at 65% of HRR for 25 min in weeks 1-2, 65–75% HRR for 35 min in weeks 3-5, and at 75–85% HRR for 40 min in weeks 6-8.	Indoor Treadmill Aerobic Exercise (ITAE): warm-up at 20% reserve heart rate (HRR); aerobic exercise on treadmill at 65% HRR for 25 min in weeks 1-2, 65–75% HRR for 35 min in weeks 3-5, and at 75–85% HRR for 40 min in weeks 6-8.	3 sessions per week for 8 weeks	Weight<br>BMI<br>Body fat %<br>VO_{2max}<br>Serum orexin A<br>RPE (Borg Category Ratio scale 1-10)<br>Mean distance during session (km)
Özbay *et al.*, 2020<br><br>Turkey, setting NR	Between subjects, controlled trial, randomization NR, parallel group	Healthy males<br><br>N=32 (16 per group)<br><br>Age (y): OE: 21.25 ± 2.40; IE: 22.62 ± 1.58	Outdoors at -5°C–5°C environmental temperature	Indoors at 21°C–25°C	40-min aerobic running exercises at a standard velocity of 65%–70% of maximum heart rate (50%–55% VO_{2max})	40-min aerobic running exercises at a standard velocity of 65%–70% of maximum heart rate (50%–55% VO_{2max})	4 days per week for 18 weeks	Serum irisin<br>Serum adropin<br>HDL-cholesterol<br>LDL-cholesterol<br>Total cholesterol<br><br>Control variables:<br>Attendance<br>Weight<br>Body fat %
Zhou *et al.*, 2020<br><br>China, nursing home	Between subjects, randomized controlled trial, parallel group	Stable-condition, independently ambulating nursing home residents<br><br>N=22 (11 per group)<br><br>Age (y): OE: 79.5 ± 2.1; IE: 80.8 ± 5.2<br><br>Sex: 86.4% male, 13.6% female	A 64 m multisurface terrain path in an outdoor therapeutic garden, part of the atrium garden of the rehabilitation center in the nursing home. An enriched environment was formed with fresh air, abundant sunlight, lush vegetation, and intermittent birdsong. The path was constructed with	A solid ground terrain in an indoor public hall of a residential building in the nursing home. A red square with a side length of 4 m was pasted on the floor of the hall that was flat and anti-slip and provided firm support. Sofa and seats placed beside the square line could be used to rest. The natural	Same program for both groups: agility, balance, and strength training, including 9 exercises: forward walking, backward walking, sideways walking, heel-to-toe walking, crossover side step, high-knee walking, squats, single-leg squats, and moving from the heels to the toes while standing. The subjects performed a set of	Same program for both groups: agility, balance, and strength training, including 9 exercises: forward walking, backward walking, sideways walking, heel-to-toe walking, crossover side step, high-knee walking, squats, single-leg squats, and moving from the heels to the toes while standing. The subjects performed all of	~30 min, 5 days per week for 3 weeks	2-min walk test<br>Timed 10-meter walk test<br>Timed Up and Go test<br>Multisurface terrain walk test<br>Single-leg standing test with eyes open and with eyes closed<br>Closed-cycles test

	various materials, including grassland (uneven texture, firm support), plastic cement (even texture, medium support), sand (fine texture, loose support), gravel (coarse texture, medium support), and unfixed pebbles (uneven texture, loose support). Supporting facilities included double-layer railings, rest seats, and signage.<br><br>Date: December 2019–January 2020<br><br>Every session in the morning	light from the window on one side of the hall combined with the auxiliary illumination of ceiling lamps in the hall ensured that there was enough light during training.<br><br>Date: December 2019–January 2020<br><br>Every session in the morning	exercises on every type of surface material (grass, sand, gravel, pebble, and plastic), and trained with their usual shoes.<br><br>Equivalent distances were travelled outdoors and indoors.	the exercises on the firm surface, and trained with their usual shoes.<br><br>Equivalent distances were travelled outdoors and indoors.	

*Duranso *et al.* (2018): Baseline parent sample had N=235, including non-intervention control groups; groupwise baseline sample sizes NR. After drop-outs and removal of missing data (n=50), post-test parent sample had N=185, including n=87 subtotal for OE and IE. Age and sex reported only for post-test parent sample.

§Irandoust & Taheri (2017): Age reported only for parent sample.

Table S4. Reported results of individual studies. Acronyms: SD: Standard Deviation; NS: Not Significant; NR: Not Reported; EMM: Estimated Marginal Mean; SE: Standard Error; OE: Outdoor Exercise; IE: Indoor Exercise.

**Author, Date**	**Outcome & Measure** *Subscale*	**Results**				**{Statistical Analyses} Reported Finding**
		**Pre**		**Post**		
		**Mean**	**SD**	**Mean**	**SD**	
**Abdel-Rahman and Magdy, 2014**						{unclear: RM-ANOVA or ANCOVA; post-hoc T-tests}
	**Flexibility**<br>Sit and Reach Test (units NR)					Between group:<br>Post-trial: OE > IE (p ≤ 0.05)<br>Within group:<br>OE: Post > Pre (p ≤ 0.05)<br>IE: Post > Pre (p ≤ 0.05)
	OE (n=10)	17.18	0.86	18.14	1.01	
	IE (n=10)	17.25	1.02	17.66	0.98	
	**Agility**<br>Shuttle Run Test (units NR)					Between group:<br>Post-trial: OE < IE (p ≤ 0.05)<br>Within group:<br>OE: Post < Pre (p ≤ 0.05)<br>IE: Post < Pre (p ≤ 0.05)
	OE (n=10)	7.15	2.18	7.03	0.14	
	IE (n=10)	7.12	0.21	7.06	0.32	
**Anandh, Varadharajulu, & Alate, 2020**						{Independent samples T-test between post-trial group means}
	**Social Activity Self-Efficacy**<br>Scale NR					Between group:<br>Post-trial: OE < IE (p = 0.0037)
	OE (n=38)			44.42	7.354	
	IE (n=38)			50.08	9.030	
	**Physical Function**					
	Timed 10-Meter Walk Test (units NR)					Between group:<br>Post-trial: OE < IE (p = 0.0151)
	OE (n=38)			14.48	1.569	
	IE (n=38)			15.39	1.621	
	Timed Up and Go Test (units NR)					Between group:<br>Post-trial: NS (p = 0.3378)
	OE (n=38)			12.90	1.003	
	IE (n=38)			13.16	1.28	
**Calogiuri, Nordtug, & Weydahl, 2015 [Intervention study]**						{Linear mixed-effects modelling}
	**EMM ± SE for all results**					

**Perceived Restorativeness Scale**				
Fascination Subscale				Group difference: OE > IE (p < 0.001)
5-Item Average (0-10)				
	OE (n=7)	6.54	0.42	
	IE (n=7)	1.90	0.42	
Being Away Subscale				Group difference: OE > IE (p < 0.001)
2-Item Average (0-10)				
	OE (n=7)	7.75	0.40	
	IE (n=7)	4.82	0.40	
**Enjoyment: Biking**				Group difference: OE > IE (p = 0.02)
Single Item (0-10)				
	OE (n=7)	6.86	0.69	
	IE (n=7)	4.29	0.69	
**Enjoyment: Strength**		NR		NS
Single Item (0-10)				
**Physical Activity Affective Scale**				
Positive Affect Subscale		NR		Group difference: OE > IE (reported with no data)
3-Item average (0-4)				
Tranquility Subscale		NR		NR
3 Item average (0-4)				
Negative Affect Subscale		NR		NR
3-Item average (0-4)				
Fatigue Subscale		NR		NR
3-Item average (0-4)				
**Intention to Exercise in Future**				Group difference: OE > IE (p < 0.01)
3-Item Average (-3-+3)				
	OE (n=7)	2.9	0.38	
	IE (n=7)	1.11	0.46	
**Follow-up Weekly Exercise**				Group difference: OE > IE (p < 0.01)
Leisure Time Exercise Questionnaire (hours/week)[i]				
	OE (n=6)	4.44	0.25	
	IE (n=5)	2.56	0.30	
**Follow-up Exercise Behaviour**				
Biking indoors				NS
Single Item (1-3)				
	OE (n=6)	1.45	0.31	
	IE (n=5)	1.00	0.41	
Biking in nature				NS
Single Item (1-3)				
	OE (n=6)	2.00	0.25	
	IE (n=5)	1.15	0.29	
Exercising with rubber bands				NS
Single Item (1-3)				
	OE (n=6)	1.78	0.19	
	IE (n=5)	1.02	0.27	

**Calogiuri *et al.*, 2016**		**EMM ± SE for all results**		{Linear mixed-effects modelling}
	**Perceived Restorativeness Scale**			
	Fascination Subscale<br>5-Item Average (0-10)			Group Difference: OE > IE (p < 0.001)
	OE (n=7)	6.54	0.42	
	IE (n=7)	1.90	0.42	
	Being Away Subscale<br>2-Item Average (0-10)			Group Difference: OE > IE (p < 0.001)
	OE (n=7)	7.75	0.40	
	IE (n=7)	4.82	0.40	
	**Physical Activity Affective Scale**			
	Positive Affect Subscale<br>3-Item Average (0-4); Baseline Corrected			Group Difference: OE > IE (p = 0.001)
	OE (n=7)	3.50	0.11	
	IE (n=7)	2.79	0.1	
	Positive Affect Subscale<br>3-Item Average (0-4); Pre-Exercise Corrected			NS
	OE (n=7)	3.31	0.14	
	IE (n=7)	2.91	0.13	
	Positive Affect Subscale<br>3-Item Average (0-4); 10-Week Follow-up[h]			Group Difference: OE > IE (p = 0.02)
	OE (n=6)	3.21	0.10	
	IE (n=5)	2.82	0.11	
	Tranquility Subscale<br>3-Item Average (0-4)			NS
	OE (n=7)	3.42	0.13	
	IE (n=7)	3.41	0.13	
	Tranquility Subscale<br>3-Item Average (0-4); 10-Week Follow-up[h]			NS
	OE (n=6)	3.15	0.15	
	IE (n=5)	3.00	0.17	
	Negative Affect Subscale<br>3-Item Average (0-4)			Excluded due to "very poor normality at all timepoints"
	OE (n=7)	NR	NR	
	IE (n=7)	NR	NR	
	Fatigue Subscale<br>3-Item Average (0-4)			Excluded due to "very poor normality at all timepoints"
	OE (n=7)	NR	NR	
	IE (n=7)	NR	NR	
	**Blood Pressure**			
	Systolic (mmHg)			NS

	OE (n=6)			119.99	2.85	
	IE (n=6)			122.28	2.83	
	Diastolic (mmHg)					Group Difference: OE < IE (p = 0.05)
	OE (n=6)			72.96	1.71	
	IE (n=6)			78.28	1.70	
	**Cortisol**					
	Absolute Cortisol Concentration					NS
	Serum (nmol/L)					
	OE (n=7)			362.92	21.87	
	IE (n=7)			343.78	20.20	
	Cortisol Awakening Response					Group Difference: OE < IE (p = 0.04)
	Salivary (AUC w.r.t. Increase)					
	OE (n=7)			10.56	27.93	
	IE (n=7)			67.23	23.79	
	Cortisol Awakening Response					NS
	Salivary (AUC w.r.t. Ground)					
	OE (n=7)			551.01	53.78	
	IE (n=7)			583.10	520.81	
**Duranso, 2018**						{One-way RM-ANOVA; Paired samples T-tests, by group, for each outcome}
	**Exercise-Specific Approach Motivation**					
	Achievement Goals Questionnaire for Sport					
	Mastery Approach Subscale					NR for interaction
	3 Items (each 0-7)					
	OE (n=48)	18.21	2.07	18.73	1.80	Within group: NS
	IE (n=39)	18.49	1.73	18.69	2.07	Within group: NS
	**Exercise-Specific Self-Efficacy**					NR for interaction
	Exercise Self-Efficacy Scale					
	10 Items (each 1-4)					
	OE (n=48)	31.10	5.29	34.02	4.25	Within group: Post > Pre (p = 0.01)
	IE (n=39)	30.87	5.38	34.46	4.82	Within group: Post > Pre (p = 0.00)
	**General Self-Efficacy**					NR for interaction
	New Generalized Self-Efficacy Scale					
	8 Items (each 1-5)					
	OE (n=48)	31.17	4.49	34.13	3.34	Within group: Post > Pre (p < 0.001)
	IE (n=39)	32.26	3.78	34.36	3.31	Within group: Post > Pre (p < 0.001)
	**Global Approach Motivation**					NR for interaction
	Revised Appetitive Motivation Scale					
	14 Items (each 1-5)					
	OE (n=48)	51.68	5.90	55.01	5.75	Within group: Post > Pre (p < 0.001)
	IE (n=39)	55.47	5.60	57.70	5.43	Within group: Post > Pre (p = 0.01)

**Irandoust & Taheri, 2017**							{RM-ANOVA; Tukey post-hoc T-tests}
	**Beck Depression Inventory**[n]<br>21 Items (each with 0-3 scale)						Between group: NS<br>Within group:<br>Significant decrease in both groups (p=0.001)
		OE (n = 15)	35.9	2.11	22.2	2.1	
		IE (n = 15)	35.9	13	26	1.8	
	**Anthropometry**						
	Weight (kg)						Between group: NS<br>Within group:<br>Significant decrease in both groups (p < 0.05)
		OE (n = 15)	76.6	3.07	73.63	3.075	
		IE (n = 15)	75.94	1.83	71.66	3.73	
	BMI (kg/m^2)						Between group: NS<br>Within group:<br>Significant decrease in both groups (p < 0.05)
		OE (n = 15)	30.04	1.59	28.88	1.63	
		IE (n = 15)	30.04	1.63	27.93	1.96	
	Waist-to-Hip Ratio						Between group: NS<br>Within group:<br>Significant decrease in both groups (p < 0.05)
		OE (n = 15)	0.98	0.04	0.92	0.03	
		IE (n = 15)	0.98	0.03	0.92	0.03	
	Body Fat %						Between group: NS<br>Within group:<br>Significant decrease in both groups (p < 0.05)
		OE (n = 15)	40.7	3.35	34.13	2.8	
		IE (n = 15)	41.18	3.072	34.21	2.69	
	**Serum 25(OH) Vitamin D** (ng/mL)						Between group:<br>Post-trial: OE > IE (p < 0.05)<br>Within group:<br>Significant increase in OE only (p < 0.05)
		OE (n = 15)	<20	NR	19.2	1.9	
		IE (n = 15)	<20	NR	14.01	1.7	
**Lacharité-Lemieux, Brunelle, and Dionne, 2015**							{ *FS, FAS, RPE:*<br>2 (Setting) x 2 (Time) RM-ANOVA<br>*EFI:*<br>2 (Setting) x 3 (Time) RM-ANOVA<br>Wilcoxon signed rank test and Friedman two-way ANOVA to confirm results<br>*Adherence:*<br>Mann-Whitney U test<br>*BDI and PASE:*<br>2 (Setting) x 2 (Time) RM-ANOVA; exploratory analyses on pre-to-post changes with Student's t test }

**Affective Valence**		Week 1		Week 11		Absolute scores:
Feeling Scale (-5 – +5)						NS for Time, Group, and Interaction effects<br><br>Exercise-induced change (EC) in absolute scores from pre-exercise to during exercise (35th min of session):<br>NS for Time and Interaction effects<br>Group main effect:<br>OE > IE ($F_{1,19}$ = 5.56; p = 0.029, η_p^2 = 0.226)
Pre exercise						
	OE (n=12)	2.75	2.34	2.58	1.88	
	IE (n=11)	3.81	1.25	3.67	1.12	
During exercise						
	OE (n=12)	4.08	1.24	3.42	1.38	
	IE (n=11)	3.36	1.43	3.22	1.48	
**Affective Activation**		Week 1		Week 11		NS for absolute scores and for EC in absolute scores
Felt Arousal Scale (1-6)						
Pre exercise						
	OE (n=12)	3.58	1.24	3.42	1.16	
	IE (n=11)	3.36	1.43	3.78	0.67	
During exercise						
	OE (n=12)	4.08	1.24	4.58	0.99	
	IE (n=11)	4.1	1.60	4.78	0.67	
**Exercise-Induced Feeling Inventory**		Week 1		Week 12		
Revitalization Subscale<br>Three Item (0-12)		NR		NR		NS
Positive Engagement Subscale<br>Three Item (0-12)		NR		NR		NS for effect of Group and for Group x Time interaction<br>Time main effect: ($F_{2,13}$ = 4.07; p = 0.043; η_p^2= 0.385)<br>Post-hoc comparisons:<br>Within group:<br>IE: Week 7 < Week 1, p ≤ 0.05; Week 12 < Week 1, p ≤ 0.05
Tranquility Subscale<br>Three Item (0-12)		NR		NR		NS for Group and Time main effects<br>Group x Time interaction ($F_{2,13}$ = 7.85; p = 0.006; η_p^2 = 0.547):<br>Post-hoc comparisons:<br>Within group:<br>IE: Week 12 < Week 1 ($F_{1,5}$ = 9.24; p = 0.029; η_p^2 = 0.649)<br>OE: Week 12 > Week 1 ($F_{1,9}$ = 5.95; p = 0.037; η_p^2 = 0.398)
Physical Exhaustion Subscale<br>Three Item (0-12)		NR		NR		NS
**Adherence**						Group difference:
% Sessions Attended						OE > IE (Z = -2.69; p = 0.007) by Mann-Whitney U Test
	OE (n=12)			97		
	IE (n=11)			91		
**Self-Reported Physical Activity**						NS for Time, Group, and Interaction effects
Physical Activity Scale for the Elderly (kcal/week)[r]						Post-hoc comparisons:<br>Within group:

							OE: Post > Pre ($F_{1,11}$ = 5.19; p = 0.044; η_p^2 = 0.321)<br>IE: NS (p = 0.71)
		OE (n=12)	148.26	54.53	184.61	68.90	
		IE (n=11)	147.98	75.22	148.96	66.59	
	**Beck Depression Inventory**<br>21 Items (each with 0-3 scale)						NS for effect of Group and for Group x Time interaction<br>Time main effect: Post < Pre ($F_{1,21}$ = 7.89; p = 0.010; ω^2 = 0.273)<br>Post-hoc comparisons:<br>Within group:<br>OE: Post > Pre ($F_{1,11}$ = 5.57; p = 0.035; η_p^2 = 0.344)<br>IE: NS (p = 0.15)
		OE (n=12)	1.88	2.41	0.63	1.15	
		IE (n=11)	1.25	1.35	0.55	0.69	
**Lacharité-Lemieux & Dionne, 2016**			Mean ± SE for all results				{ *Intensity:*<br>2 (setting) × 4 (time) between-within factorial ANOVA<br>Step-down analysis: Friedman's two-way ANOVA and Mann-Whitney U test<br>*Other outcomes:*<br>2 (setting) × 2 (time) between-within factorial ANOVA<br>Step-down analysis: Wilcoxon signed-rank test and Mann-Whitney U test }
	**Anthropometry**						NS for Group x Time interaction for all anthropometric variables
	Weight (kg)						Time main effect:<br>Post < Pre, $F(1,19) = 6.55$, $p = .019$, η_p^2 = 0.256
		OE (n=12)	63.40	6.63	62.78	6.99	
		IE (n=11)	66.09	8.03	65.22	8.61	Within group:<br>IE: Post < Pre, $p \leq .05$ (Wilcoxon signed-rank test)
	BMI (kg/m²)						
		OE (n=12)	25.40	1.85	25.27	2.08	
		IE (n=11)	25.65	1.75	25.06	1.93	Within group:<br>IE: Post < Pre, $p \leq .01$ (Wilcoxon signed-rank test)
	Waist Circumference (cm)						
		OE (n=12)	87.87	8.52	85.64	8.55	Time main effect:<br>Post < Pre, $F(1,19) = 12.701$, $p = .02$, η_p^2 = .401
		IE (n=11)	92.43	6.29	88.16	6.10	Within group:<br>IE: Post < Pre, $p \leq .01$ (Wilcoxon signed-rank test)
	Fat Mass (kg)						NS
		OE (n=12)	22.38	6.14	22.1	6.39	
		IE (n=11)	25.89	5.01	25.4	5.11	
	Lean Body Mass (kg)						NS
		OE (n=12)	38.34	2.63	38.04	2.27	
		IE (n=11)	37.34	3.58	37.38	3.54	
	% Fat Mass						NS
		OE (n=12)	36.35	6.73	36.16	6.78	

	IE (n=11)	40.67	3.98	40.15	3.92	
Muscle Mass Index (kg/m[2])						Group main effect: OE > IE, $F(1,19) = 6.656$, $p = .018$, $\eta_p^2 = .259$
	OE (n=12)	15.45	1.03	15.35	0.92	Between group: OE > IE at each time point, $p \leq .01$ (Mann-Whitney U test)
	IE (n=11)	14.45	0.39	14.40	0.53	
**Maximum Oxygen Uptake** VO_{2max} (ml·kg^{-1}·min^{-1})						Time main effect: Post > Pre, $F(1,19) = 10.24$, $p = .005$, $\eta_p^2 = .345$ NS for Group and Group x Time interaction effects
	OE (n=12)	23.32	4.67	25.13	4.12	
	IE (n=11)	21.51	4.40	25.05	3.28	Within group: IE: Post > Pre, $p = .01$ (Wilcoxon signed-rank test)
**Plasma Lipids**						
Triglycerides (mmol·L^{-1})						NS
	OE (n=12)	1.06	0.43	1.07	0.49	
	IE (n=11)	1.49	0.88	1.56	1.22	
Total cholesterol (mmol·L^{-1})						Time main effect: Post < Pre, $F(1,18) = 8.14$, $p = .011$, $\eta_p^2 = .311$
	OE (n=12)	5.89	0.82	5.72	0.92	Within group: OE: Post < Pre, Z = –2.31, $p = .021$ (Wilcoxon signed-rank test)
	IE (n=11)	6.30	1.29	6.04	1.17	
Cholesterol-HDL (mmol·L^{-1})						Between group: Pre-trial: OE > IE, Z = –2.28, $p = .023$ (Wilcoxon signed-rank test)
	OE (n=12)	2.05	0.54	1.97	0.57	
	IE (n=11)	1.62	0.18	1.61	0.25	
Cholesterol-LDL (mmol·L^{-1})						NS
	OE (n=12)	3.36	0.86	3.08	1.04	
	IE (n=11)	4.11	0.97	3.64	0.81	
**Fasting glucose** (mmol·L^{-1})						NS
	OE (n=12)	4.91	0.47	4.88	0.44	
	IE (n=11)	4.91	0.74	4.79	0.33	
**Fasting insulin** (pmol·L^{-1})						NS
	OE (n=12)	37.91	24.18	37.00	22.58	
	IE (n=11)	45.33	24.12	39.82	26.35	
**Insulin Resistance** Homeostasis Model Assessment[s]						NS
	OE (n=12)	1.24	0.91	1.19	0.80	
	IE (n=11)	1.48	0.82	1.24	0.83	
**Blood Pressure**						
Systolic (mmHg)						Time main effect: Post < Pre, $F[1,18] = 10.92$, $p = .004$, $\eta_p^2 = .365$
	OE (n=12)	123.17	10.34	113.92	10.14	Within group: OE: Post < Pre, $p = 0.013$

	IE (n=11)	112.05	14.80	108.75	13.91	
Diastolic (mmHg)						Time main effect:<br>Post < Pre, F[1, 18] = 9.19, p = .007, η_p^2 = .326
	OE (n=12)	77.13	4.96	71.46	5.82	Within group:<br>OE: Post < Pre, p = 0.020
	IE (n=11)	71.17	9.43	68.91	10.45	
**Muscle Strength**<br>One-Repetition Maximum (1-RM)						
Leg Press (lbs)						NS for interaction<br>Time main effect:<br>Post > Pre, F(1,19) = 9.57, p = .006, η_p^2 = .335
	OE (n=12)	155.83	43.37	187.92	58.83	Within group:<br>OE: Post > Pre, p = 0.016
	IE (n=11)	135.91	49.94	159.55	68.06	Within group:<br>IE: Post > Pre, p = 0.021
Bench Press (lbs)						Time main effect:<br>Post > Pre, F[1, 19] = 10.49, p = .004, η_p^2 = .356
	OE (n=12)	53.33	17.36	63.33	17.56	Within group:<br>OE: Post > Pre, p = 0.006 (Wilcoxon signed-rank test)
	IE (n=11)	43.96	18.14	51.59	15.34	
Lat Pull Down (lbs)						Time main effect:<br>Post > Pre, F[1, 18] = 5.66, p = .029, η_p^2 = .239
	OE (n=12)	76.67	10.73	83.75	12.27	Within group:<br>OE: Post > Pre, p = 0.017
	IE (n=11)	71.82	19.53	75.01	15.33	
**Muscular Endurance**<br>Maximum Repetitions of 70% 1-RM						
Leg Press (lbs)						NS for interaction<br>Time main effect: Post > Pre, F(1,19) = 41.05, p < .001, ηp2 = .684
	OE (n=12)	16.50	5.02	28.33	9.59	Within group:<br>OE: Post > Pre, p < .001 (Wilcoxon signed-rank test)
	IE (n=11)	17.27	5.24	27.45	11.55	Within group:<br>IE: Post > Pre, p = .001
Bench Press (lbs)						Time x Group interaction: F(1,18) = 4.47, p = .049, η_p^2 = .199<br>Time main effect: Post > Pre, F(1,17) = 8.80, p = .009, η_p^2 = .328
	OE (n=12)	9.00	2.83	15.17	3.67	Within group:<br>OE: Post > Pre, p = 0.003 (Wilcoxon signed-rank test)<br>Between group:<br>Post-trial: OE > IE, p ≤ .05
	IE (n=11)	9.92	4.10	11.90	2.51	
Lat Pull Down (lbs)						Time main effect:<br>Post > Pre, F(1,18) = 8.78, p = .008, η_p^2 = .341
	OE (n=12)	13.33	5.18	18.91	8.46	Within group:

							OE: Post > Pre, $p = 0.023$
		IE (n=11)	14.55	5.07	18.09	5.43	

Study	Outcome	Group	Change Score	95% CI Lower	95% CI Upper		{Generalized Estimating Equations}
**Miller *et al.*, 2020**	**Exercise Motivation**<br>Behavioral Regulation in Exercise Questionnaire-2						
	Overall Score<br>19 Items (-23-39)						NS
		OE	0.10	-1.86	2.06		n=12 (total over both groups)
		IE	0.22	-1.00	1.45		
	Intrinsic Subscale<br>4 Items (each 0-4)						NS
		OE	-0.08	-0.32	0.16		n=12 (total over both groups)
		IE	0.20	0.04	0.37		
	Identified Subscale<br>3 Items (each 0-4)						NS
		OE	0.08	-0.07	0.22		n=13 (total over both groups)
		IE	-0.03	-0.36	0.30		
	Introjected Subscale<br>4 Items (each 0-4)						NS
		OE	0.03	-0.29	0.34		n=12 (total over both groups)
		IE	0.19	-0.22	0.61		
	Extrinsic Subscale<br>4 Items (each 0-4)						Between groups:<br>OE > IE ($p < 0.05$)
		OE	-0.13	-0.47	0.22		n=12 (total over both groups)
		IE	0.39	0.02	0.75		
	Amotivation Subscale<br>4 Items (each 0-4)						NS
		OE	0.00	-0.40	0.40		n=12 (total over both groups)
		IE	-0.16	-0.33	0.02		
	**Psychological Need Satisfaction in Exercise**						
	Autonomy Subscale<br>Single Item (0-6)						NS
		OE	-0.31	-0.70	0.09		n=12 (total over both groups)
		IE	0.10	-0.17	0.37		
	Competence Subscale<br>Single Item (0-6)						NS
		OE	0.23	-0.24	0.70		n=13 (total over both groups)
		IE	0.01	-0.20	0.23		
	Relatedness Subscale<br>Single Item (0-6)						NS
		OE	0.30	-0.22	0.82		n=10 (total over both groups)
		IE	-0.12	-0.56	0.32		

	**Fatigue**<br>Fatigue Scale-Adolescent<br>13 Item (13-65)						
	General						NS
		OE	1.08	-0.51	2.67		n=10 (total over both groups)
		IE	0.00	-1.82	1.82		
	In-Session						NS
		OE (n=NR)	NR	NR	NR	NR	
		IE (n=NR)	NR	NR	NR	NR	
	**Moderate to Vigorous Physical Activity**<br>Actigraph GT3x accelerometer<br>Habitual (minutes per week)						NS
		OE	-2.9	-13.0	7.2		n=10 (total over both groups)
		IE	-6.1	-18.4	6.3		
**Moslehi, Moslehi, & Khalvati, 2019**							{One-way ANOVA with Tukey post-hoc tests to compare pre-post changes between groups; effect sizes reported, presumably as Cohen's *d* (reviewer judgement)}
	**Anthropometry**						
	Weight (kg)						Between group: OE↓ > IE↓<br>Effect size = 0.48
		OE (n=10)	66.68	4.1	61.52	3.7	
		IE (n=10)	68.61	3.89	65.45	4.11	
	BMI (kg/m^2)						Between group: OE↓ > IE↓<br>Effect size = 0.27
		OE (n=10)	27.94	2.06	25.77	1.71	
		IE (n=10)	28.27	2.04	26.96	1.84	
	Body Fat %						Between group: OE↓ > IE↓<br>Effect size = 0.47
		OE (n=10)	31.9	3.2	28.51	2.03	
		IE (n=10)	32.57	3.02	31.52	3.08	
	**Maximum Oxygen Uptake**<br>VO_{2max} ($ml \cdot kg^{-1} \cdot min^{-1}$)						Between group: NS<br>Effect size = 0.06
		OE (n=10)	30.94	1.62	32.79	1.73	
		IE (n=10)	30.71	2.01	33.13	2.19	
	**Serum orexin A** (ng/mL)						Between group: OE↑ > IE↑<br>Effect size = 0.37
		OE (n=10)	49.28	3.62	60.88	5.72	
		IE (n=10)	51.49	3.62	56.9	2.77	
**Özbay *et al.*, 2020**							{Repeated-measures two-way ANOVA with Bonferroni post-hoc tests for main outcomes; within-group paired T-tests to assess pre-post changes for weight and body fat}

	**Serum Irisin** (ng/mL)								Between group: NS
		OE (n=16)	39.76	16.98		41.21	18.65		
		IE (n=16)	40.28	11.29		34.13	14.71		Within group: Post < Pre, $p < 0.05$, $\eta^2 = 0.506$
	**Serum Adropin** (ng/L)								Between group: NS
		OE (n=16)	1002.58	531.05		558.18	343.44		Within group: Post < Pre, $p < 0.05$, $\eta^2 = 0.711$
		IE (n=16)	915.75	453.08		799.16	405.60		
	**Plasma Cholesterol**								
	HDL-cholesterol (mg/dL)								Between group:<br>Post-test: OE > IE, $p < 0.05$
		OE (n=16)	49.01	8.20		56.43	9.52		Within group: Post < Pre, $p < 0.05$, $\eta^2 = 0.909$
		IE (n=16)	44.15	5.01		46.87	5.52		Within group: Post < Pre, $p < 0.05$, $\eta^2 = 0.693$
	LDL-cholesterol (mg/dL)								Between group: NS
		OE (n=16)	84.01	16.71		83.01	11.31		
		IE (n=16)	97.76	35.45		97.37	22.16		
	Total cholesterol (mg/dL)								Between group: NS
		OE (n=16)	151.82	19.15		159.05	18.11		
		IE (n=16)	162.05	42.28		167.12	24.19		
	**Anthropometry**								
	Weight (kg)								Between group: NS
		OE (n=16)	69.00	4.86		69.21	4.88		
		IE (n=16)	70.87	6.91		71.04	6.82		
	Body Fat %								Between group: Differences NR
		OE (n=16)	9.26	2.07		9.08	2.08		Within group: Post < Pre, $p = 0.0001$
		IE (n=16)	9.16	1.91		8.98	1.73		Within group: Post < Pre, $p = 0.028$
**Zhou *et al.*, 2020**			Median	25th and 75th percentiles		Median	25th and 75th percentiles		{Changes in outcomes were compared between groups using the Mann–Whitney U-test and within groups using the Wilcoxon signed-rank test. Pre- and post-test values and change scores all reported.}
	**Walking Ability**								
	2-Min Walk Test (m/s)								Between group: OE ↑ > IE ↑
		OE (n=11)	110	91	135	120	95	136	
		IE (n=11)	120	112.8	120	125	115	135	
	Timed 10-Meter Walk Test (m/s)								Between group: OE ↑ > IE ↑
		OE (n=11)	0.87	0.57	1.06	0.94	0.64	1.09	
		IE (n=11)	0.84	0.76	1.18	0.94	0.81	1.18	
	Multisurface Terrain Walk Test (s)								Between group: OE ↑ > IE ↑
		OE (n=11)	92.4	63.1	126.6	76.9	58.6	108.2	
		IE (n=11)	69	61	81	63.6	58.7	75.3	
	**Balance**								
	Timed Up and Go Test (s)								NS
		OE (n=11)	13.1	10.7	19	12.9	9.6	16.1	
		IE (n=11)	12.1	8.1	13.3	10.6	8.3	12.4	
	Single-Leg Standing Test with Eyes Open (s)								NS

OE (n=11)	2.8	1.5	5.3	3.7	3.1	8.3	
IE (n=11)	3.7	3.1	9	4.1	3.1	22.9	
Single-Leg Standing Test with Eyes Closed (s)							NS
OE (n=11)	2.6	1.2	3	2.8	2.5	4	
IE (n=11)	2.5	1.6	3.2	2.9	2.4	3.2	
Closed-Cycles Test (s)							NS
OE (n=11)	5.6	4.1	8	9.2	5.5	11.9	
IE (n=11)	7.6	5.7	14.2	8.6	5.9	16.5	

Notes on outcome measures (in order of appearance):

Perceived Restorativeness Scale: each item rated from 0 (absolutely disagree) to 10 (absolutely agree) (Hartig, *et al*., 1997)

Enjoyment: rated from 0 (not enjoyable at all) to 10 (absolutely enjoyable)

Physical Activity Affective Scale: 4 subscales, each with 3 items measured on a 5-point Likert scale from 0 (strongly disagree) to 4 (strongly agree) (Lox *et al*., 2000)

Intention to exercise in future: 3 items referring to exercise behavior in the next 10 weeks; each item rated on 7-point scale: –3: Strongly disagree, 0: Neither agree nor disagree, +3: Strongly agree

Leisure Time Exercise Questionnaire: adjusted to measure total hours of exercise per week (Godin & Shephard, 1985)

Follow-up exercise behaviour: values expressed as frequency: 1: Seldom/never, 2: Sometimes, and 3: Often/daily

Cortisol AUC: Area Under the Curve with respect to increase and with respect to ground, as described by Pruessner *et al.* (2003)

Achievement Goals Questionnaire for Sport, Mastery Approach subscale: 3 items, each rated from 1 (not at all like me) to 7 (completely like me) (Conroy *et al*., 2003)

Exercise Self-Efficacy Scale: 10-item scale, each item rated on a 4-point Likert scale ranging from 1 (not at all true) to 4 (always true) (Kroll *et al*., 2007)

New Generalized Self-Efficacy Scale: 8-item scale, each item rated from 1 (strong disagreement) to 5 (strong agreement) (Chen *et al*., 2001)

Appetitive Motivation Scale (revised version): 14-items, each rated from 1 (strongly disagree) to 5 (strongly agree) (Leone *et al*., 2011)

Beck Depression Inventory: 21 items, each rated from 0 to 3 in terms of increasing intensity. Total score from 21 items reported by Irandoust & Taheri, 2017; average score per item reported by Lacharité-Lemieux, Brunelle, and Dionne, 2015.

Feeling Scale: rating of how one presently feels from minus 5 (very bad) to 5 (very good)

Felt Activation Scale: rating of perceived activation from 1 (low arousal) to 6 (high arousal)

Exercise-Induced Feeling Inventory: 4 affective states, each with 4 subscale items rated from 0 (do not feel) to 4 (feel very strongly) (Gauvin & Rejeski, 1993)

Physical Activity Scale for the Elderly: "measures self-reported frequency, intensity, and duration of any physical activity performed in one's leisure, work, or exercise time during the previous 7 days" (Washburn *et al*., 1993)

Homeostasis Model Assessment for insulin resistance was calculated by the equation: (fasting insulin concentration [UI/ml] × fasting glucose concentration [mmol/L]) / 22.5 (Matthews *et al*., 1985).

Behavioral Regulation in Exercise Questionnaire-2: 19-item survey assessing exercise motivation (range: −23 to 39; higher score indicates greater intrinsic motivation), with subscale scores for each Self-Determination Theory class of motivation: intrinsic, identified, introjected, extrinsic motivation, and amotivation (range: 0 to 4; higher score indicates greater endorsement of motivation class). (Markland and Tobin, 2004)

Psychological Need Satisfaction in Exercise: 18-item survey with 3 subscales - perceived autonomy, competence, and relatedness – each ranging from 0 to 6 (higher values indicate greater endorsement of the feeling). (Wilson *et al*., 2006)

Moderate to vigorous physical activity (MVPA): measured using Actigraph GT3x accelerometers worn on the hip for 7 days at baseline, 2 weeks after the first two exercise sessions, and 2 weeks after the last two exercise sessions (Habitual MVPA) or during exercise sessions (In-session MVPA).

Fatigue Scale-Adolescent: 13-item survey (range: 13-65; higher score indicates greater fatigue) (Mandrell *et al*., 2011)

Serum Orexin A (OXA): Blood samples were collected 24 h before and after the exercise period following 12 h of overnight fasting. Means of triplicate serum OXA measured according to the protocol of the human OXA ELISA Kit (Cat# EK-003-30) Phoenix company.

Irisin and adropin concentrations were measured with a commercially available enzyme immunoassay kit (Human irisin ELISA kit 201-12-5328; Human adropin ELISA kit 201-12-2015, SunRed, China)

10m Walk Test: Subjects were asked to walk at a comfortable pace; walking speed recorded over middle 6 m of 10 m walkway; best of 3 trials.

2-Min Walk Test: Subjects were asked to walk as fast as possible for 2 min; distance recorded as best of 2 trials.

Multisurface Terrain Walk Test: time to walk along outdoor multisurface terrain at a comfortable speed; best of 2 trials

Single-Leg Standing Test with Eyes Open/Closed: time standing on dominant leg with eyes open/closed for as long as possible; best of 3 trials

Closed-Cycles Test: Subjects stepped in place in the center of the circle (40 cm diameter) with their eyes closed at a rate of 120 steps per minute with a metronome. Subjects stopped once a foot was out of the loop or touched the edge of the circle. Best of 3 trials.
